# Maternal care in rural China: a case study from Anhui province

**DOI:** 10.1186/1472-6963-8-55

**Published:** 2008-03-10

**Authors:** Zhuochun Wu, Kirsi Viisainen, Xiaohong Li, Elina Hemminki

**Affiliations:** 1 Department of Health Statistics and Social Medicine, Fudan University School of Public Health, 138 Yi Xue Yuan Road, Shanghai 200032, P.R. China; 2 Department of Public Health, University of Helsinki, Helsinki, Finland; 3 National Research and Development Centre for Welfare and Health (STAKES), P.O. Box 220, 00531 Helsinki, Finland

## Abstract

**Background:**

Studies on prenatal care in China have focused on the timing and frequency of prenatal care and relatively little information can be found on how maternal care has been organized and funded or on the actual content of the visits, especially in the less developed rural areas. This study explored maternal care in a rural county from Anhui province in terms of care organization, provision and utilization.

**Methods:**

A total of 699 mothers of infants under one year of age were interviewed with structured questionnaires; the county health bureau officials and managers of township hospitals (n = 10) and county level hospitals (n = 2) were interviewed; the process of the maternal care services was observed by the researchers. In addition, statistics from the local government were used.

**Results:**

The county level hospitals were well staffed and equipped and served as a referral centre for women with a high-risk pregnancy. Township hospitals had, on average, 1.7 midwives serving an average population of 15,000 people. Only 10–20% of the current costs in county level hospitals and township hospitals were funded by the local government, and women paid for delivery care. There was no systematic organized prenatal care and referrals were not mandatory. About half of the women had their first prenatal visit before the 13th gestational week, 36% had fewer than 5 prenatal visits, and about 9% had no prenatal visits. A major reason for not having prenatal care visits was that women considered it unnecessary. Most women (87%) gave birth in public health facilities, and the rest in a private clinic or at home. A total of 8% of births were delivered by caesarean section. Very few women had any postnatal visits. About half of the women received the recommended number of prenatal blood pressure and haemoglobin measurements.

**Conclusion:**

Delivery care was better provided than both prenatal and postnatal care in the study area. Reliance on user fees gave the hospitals an incentive to put more emphasis on revenue generating activities such as delivery care instead of prenatal and postnatal care.

## Background

The appropriate care of pregnant women during the prenatal, delivery and postnatal periods in order to identify risks of adverse events for both the women and their fetuses or newborn children (i.e. maternal care), is considered a priority in most health care systems [1]. In China, a systematic maternal care program was introduced in the 1980s and its utilisation and outcomes have been the target of many studies [2-8]. However, only a few of these studies are in English [3,4,9-12]. The studies showed that the utilization of maternal care increased and that perinatal and maternal health indicators have improved since the early 1980s [3,7,9,13-17].

The results of national household surveys showed that the proportion of pregnant women with the first prenatal care visit before 13 weeks of gestation increased from 19.9% to 58.1% and hospital delivery rate increased from 37.6% to 73.7% between 1993 and 2003 [7]. According to the results of national maternal and infant mortality surveillance, maternal mortality rate (MMR) fell from 94.7 to 53.4 per 100000 live births between 1989 and 2000 [14] and infant mortality rate (IMR) decreased from 37.9 per 1000 live births in 1982 to 32.5 per 1000 in 1998 [15]. In the study province MMR fell also from 62 to 50 per 100000 live births and IMR decreased from 33 to 29 per 1000 live births between 1995 and 1999 [18].

However, socioeconomic and geographic inequalities have remained. There were great differences in the use of maternal care between richer and poorer regions and between urban and rural areas, as well as in maternal and infant health status. In 1997, in urban areas, women had on average 6.4 prenatal visits in comparison to 3.2 visits in rural areas. Furthermore, while 92% of urban women gave birth in hospitals and 61% attended postnatal care, only 41% and 50% of rural women did so, respectively [19].

In 2000, MMR in urban and rural areas were 28.9 and 67.2 per 100,000 live births. MMR in richer east coast area was below 21.2 while in poorer remote west the MMR reached as high as 114.9 per 100,000 live births [16]. IMR in urban and rural areas was 25.8 and 37.0 per 1,000 live births respectively [17]. Provincial or county level studies have showed that the reported MMR and IMR from census or surveillance data were underestimated and that the underreporting rate varied: that of MMR from less than 10% to about 30% while that of IMR from 10% to 50% [20,21]. No nation-wide study has been done to evaluate the quality of the data.

The studies on prenatal care in China have focused on the timing and frequency of prenatal care [12,19,22] and relatively little information can be found on how maternal care has been organized and funded, and the actual content of the prenatal visits, especially in the less developed rural areas. The purpose of the study is to explore the maternal care in rural China in terms of care organization, provision and utilization.

## Methods

The study was conducted in a rural county of 900,000 people in Anhui province, in eastern China. The total area of the county is almost 3,000 square kilometres, mostly of flatland. The majority of the population of the county is engaged in farming. In terms of national GDP rankings, the study county is typical of less developed rural areas in China. This paper is based on the experiences of a community-based randomised control trial on prenatal care. Twenty townships (10 interventions and 10 control) out of a total of 55 were selected – on the basis of having sufficient health facilities and staff – for a controlled trial on the introduction of systematic prenatal care in the area [23]. The purpose of this randomized trial was to evaluate the effects of prenatal care on infant and maternal outcomes in the specific context of rural China, as well as to describe the process of conducting such a trial using community resources. This paper is based on the knowledge, attitudes and practices survey (KAP survey) data from the 10 townships used as controls. In addition, the data of the interviews of local government officials, interviews of health care managers, observations of service providers and local government statistics were used.

### Community-based household survey of service users

Each township in the study county was administratively divided into 6–15 villages. About 10, 20 and 30 percent of villages in ten control townships were randomly selected to be surveyed in October- December in 2001, 2002 and 2003, respectively. All women who had given birth within 12 months prior to the three KAP survey periods in these villages were approached for an interview. During that time, there were 722 women who were eligible for the study according to the records kept by the local family planning system.

In the villages, the mothers of infants aged 0–12 months were identified according to lists provided by the family planning system and were asked to participate in the interviews. The interview was conducted at the respondent's home and was based on a structured questionnaire. If the woman was not at home at the time of the survey, the father or some other family member responded on her behalf. Altogether, the survey gathered data on 699 (out of 722) women, 90% of respondents being the mothers themselves. Three percent of the sample did not provide useful data: 1 (0.14%) refused, 15 families (2.1%) were out of village, 7 (1.0%) for other reasons. Women with dead infants were not approached for interviews.

Interviewers were recruited among the local health workers in the townships. They were trained to conduct interviews by a researcher (Wu Z.) from Fudan University.

The structured questionnaire used in the survey included 60 questions covering infant outcomes, women's knowledge and attitudes, and practices relevant to prenatal, delivery, postnatal care and health, including infant care and breastfeeding. Only the questions concerning prenatal, postnatal and delivery care were used in this particular study.

### Interviews and observations of health care providers and local government statistics

The director and vice director of the county health bureau, the directors of the county maternal and child health care station, the directors of the county hospital and the directors of the 10 study township hospitals were interviewed in their offices with a semi-structured questionnaire concerning the provision and organization of services, the characteristics of the township hospital, the availability of equipment and staff, the funding of the hospital and the level of providers' income in mid-2000 and mid-2003 by one of the researchers from Fudan University (Li X.).

Direct observations of care providers and facilities were done ad hoc by the researchers from both the Fudan University and the Finnish National Research and Development Centre for Welfare and Health (STAKES) during field visits and systematically by the field research assistant who observed the implementation of maternal care trial in all the study townships and reported her findings to the researchers regularly. The observation activities included site visits to all study township hospitals and family planning services stations, to all county level hospitals including maternal and child health care institutes, and to some randomly selected village family planning posts and health clinics. The observations covered study settings, activities, and services provided by both the health care and the family planning facilities. The field research assistant visited each township at least once every month between 2000 and 2003. The researchers from Fudan University visited each of the above-mentioned institutes at least once during 2000 and 2003. The researchers from STAKES visited a selection of townships every year between 2000 and 2003. Some births occurred in private facilities but the county health authorities did not allow the researchers to visit such facilities.

The statistics of health facilities and obstetrical staff were collected from the county health bureau. The document "Management method of systematic maternal care in rural areas" issued by the Ministry of Health of China in 1989 [24] was used to compare the provision and utilization of maternal care in the study townships with the national norms. This document gives norms for the management of maternal care at village, township and county levels by health care providers and recommends the content, number and timing of prenatal and postnatal care and gives advices on the referral and delivery services; the 1989 version was the most recent one at the time of the study. The above mentioned document does not cover the issues of financing of maternal care or whether the care should be free for users. There were no official recommendations for the number of health care professionals per population.

## Results

The results of first three sections below are from interviews of health care providers and from local government statistics and observations. The results of last two sections below are from the community based household interview of users.

### Organization and provision of maternal care in the study area

The public maternal care system in the study county, like the health care system in general, was organized into three levels of care: the county, township and village level. At the county level, secondary to tertiary obstetric care was provided at two general hospitals (the county hospital and the hospital of traditional Chinese medicine) and at one specialised hospital (the maternal and child health care station). These hospitals each had 100–300 beds, 2 delivery beds, and 10–15 obstetrical staff members.

Most delivery care was taken care of in township hospitals and most prenatal care services were provided by township midwives. Township hospitals had on average 1.7 midwives serving an average population of 15,000 people. All midwives were female, from 26 to 55 years of age (mean = 36.4) with experience ranging between one and 37 years. The majority (88%) had three years of training in nursing after junior high school (9 years) and an additional six months of midwifery training.

At the village level, there were small private clinics staffed by one or two village health workers with 2–3 years of general medical training. There was no limitation for private practitioners to provide prenatal and postnatal care by the county legislation. However, they were not permitted to provide delivery care according to the county health bureau, but as some 12% of all births occurred either at home or at private clinics, some village health workers might have been involved. There was also a vast network of larger private clinics and individual practitioners in townships and cities, which were also not permitted by the county health authority to deliver babies, but may still have been involved in the births registered to have occurred outside of the public sector. If discovered to be involved in delivery care, the private practitioners would have been required to pay a fine. For the baby's family there were no consequences financially or in registering the baby.

The county level hospitals served as the referral centres for the township hospitals in the county. However, there were no formal regulations or guidelines on how the referral system should have worked. Referral to a higher level hospital depended on the individual midwife's professional judgment and on the clients' compliance. A referral was not mandatory even in critical conditions. However, women could freely seek care at the higher level hospitals if they wished and could afford the cost of travel and higher fees. Most women who went to county level hospitals did so without a formal referral.

In the study county, the county health bureau had little power over township hospitals, because township hospitals had, in the decentralisation process, become directly accountable to the township government. Some of the health bureau officials were health professionals, but that was not the case of the township's government officials.

In parallel there were three levels of family planning services: the county institute, the township service station and the village service post. The family planning institutes were in charge of implementing and supervising the national family planning policy by performing mandatory pregnancy tests regularly on all married women [25]. The family planning staff also provided maternal care consultations and performed simple obstetric examinations during the routine pregnancy test sessions. Posters related to maternal care were hung on the walls of the family planning offices. In addition to family planning services, these facilities at the county and township level could also provide delivery services. The family planning activities were by law fully funded by the county and township government and were free for the users. However, families paid for delivery care provided by family planning services.

### Financing of maternal care

In the township and county hospitals, only 10–20% of the current costs were funded by the local government (by the township government and the county government respectively) and the hospitals collected user fees to balance their budgets. The staff's salaries were dependent upon the level of activity and thereby of fee income of the hospital. The midwives' income per month varied from 36.3 to 108.8 USD (mean = 74.7 USD).

The hospitals were able to determine the user fees on their own; however, prenatal care consultations were free in most township hospitals. Only one township hospital charged 0.36 USD for a consultation. The cost of an ultrasound test ranged from 1.81 to 3.63 USD (mean = 2.65 USD), the fee for urine and haemoglobin testing were from 0.48 to 1.21 USD (mean = 0.78USD), the fee for normal delivery were from 15.72 to 36.28 USD (mean = 24.91 USD) and Caesarean sections (at county level) cost from 133.01 to 145.10 USD (mean = 139.06 USD) respectively. Farmers' annual net income per capita in the study area was about 260 USD [23], which was very close to that of national level (266 USD) and a bit higher than that of the average of the whole study province (240 USD) [26].

One township carried out a prepaid maternal care program which was organized by the township hospital and supported by the township government. In the prepaid maternal care program, the pregnant woman paid a lump sum of money to the township hospital for a package of systematic maternal care, including prenatal, delivery and postnatal care. If the child died during the delivery because of a midwife's mistakes, the hospital reimbursed some of the money back to the woman.

### Facilities and resources

Each study township hospital had a midwifery department with two to five rooms with basic delivery care equipment. In the department, the midwives saw women for prenatal and postnatal appointments. Delivery rooms were basic rooms with a simple delivery bed; all had electric lights but only 40% had running water. Manual suction pumps for infant airway clearance were available in 30% of rooms. Only two delivery rooms in the study area were equipped with air conditioners (combination heater/cooler), most used charcoal for warming up the rooms in cold winter months when temperatures can drop to minus 2–6 degrees centigrade. A total of 80% of the township hospitals had an ultrasound machine and a technician to use it, but only 50% had a scale for weighing the mother during pregnancy. Basic laboratory equipment for urine and haemoglobin tests was available in 90% of the township hospitals. Only one township hospital had an operating room and a surgeon who could perform caesarean sections. No medical records were kept in six of the 10 study township hospitals. Individual prenatal care cards were used in two out of the 10 township hospitals.

### Timing, frequency and content of prenatal care visits

The average age of the mothers interviewed was 26.3 years. 23% had two children, and very few of them had three or more. The great majority (87%) of the mothers were farmers. 79% of them had at least 6 years of education, 21% were illiterate or semi-illiterate (less than 6 years of schooling).

Most prenatal care took place in public facilities; but 15% reported having visited a private practitioner during pregnancy. The first prenatal visit took place on average at 15.7 gestational weeks (see Table 1). About half of the women had their first prenatal visit before 13 gestational weeks and 2% sought prenatal care only after 28 weeks of gestation. The timing of the first prenatal visit did not change with parity (Table 1).

**Table 1:** Timing of first prenatal care visit.

Parity	No visit	< = 12 weeks	13–27 weeks	> = 28 weeks	Total
1 (n = 450)	8.7	44.2	45.1	2.0	100.0
2+ (n = 139)	10.1	44.6	43.2	2.1	100.0
Parity unknown (n = 110)	10.9	43.6	43.6	1.8	100.0


Total	9.3	44.2	44.5	2.0	100.0

The average number of prenatal visits was 5.2. More than half of the women interviewed had 5 or more prenatal visits during their last pregnancy. Some 9% (see below) of primiparous and multiparous women had similarly made no prenatal visits (Table 1, Table 2).

**Table 2:** Frequency of prenatal care visit by parity and place of delivery (%).

	0	1–4	5+	Total
Parity				
Parity 1 (n = 450)	8.7	37.6	54.4	100.0
Parity 2+ (n = 139)	10.1	36.2	54.3	100.0
Parity unknown (n = 110)	11.8	30.0	58.2	100.0
Total (n = 699)	9.0	36.1	54.9	100.0
Place of delivery				
County hospitals (n = 175)	7.9	35.4	56.7	100.0
Township hospitals (n = 438)	4.6	37.1	58.3	100.0
Private clinics (n = 65)	33.8	33.9	32.3	100.0
At home (n = 21)	38.1	23.8	38.1	100.0

The reasons for not attending prenatal care were: felt the care was unnecessary or worthless (42%), lack of time (17%), transportation issues (15%), cost (4%), other reasons (4%), no reply (19%). The reasons given were similar among primiparous and multiparous women.

Table 3 shows the utilisation of maternal care in the study county in comparison with the current valid recommendations. Only 50% of women had received the recommended prenatal care in terms of timing, frequency and content. Blood pressure was measured on every visit for 54% of the women, 34% were measured on some occasions, and for 12% it was not measured at all. Haemoglobin measurement was performed once on 32% of women, more than once on 13% of women, and not at all on 55% of women. Ultrasound screenings during pregnancy were common: 58% had had an examination at least once. Over 70% of women received health education on maternal nutrition (77%), baby care (73%), maternal health care during pregnancy (84%) from midwives (Table 4).

**Table 3:** Comparison of the recommendations from the government and the care reported by the women.

	MOH recommendation	% complying
Timing of first prenatal visit	Before 13 weeks of gestation	44
Frequency of prenatal care visit	At least 5 times	55
Frequency of postnatal visit	At least 3 time	0
Measure of blood pressure	Every time during 5 visits	54
Haemoglobin test	At least once during the visits	45
Education on maternal nutrition	After 28 weeks of gestation	77
Education on maternal health care	During the pregnancy	84
Education on baby care	During the pregnancy	73

**Table 4:** Content of prenatal care (%).

Content of prenatal care	Parity 1 (n = 450)	Parity 2+ (n = 139)	Parity unknown (n = 110)	Total (n = 669)
Blood pressure measurement				
During every visit	54	54	54	54
Sometimes	33	36	37	34
Never	13	10	9	12
Times of haemoglobin measurement				
0	57	58	44	55
1	30	32	38	32
2+	13	10	18	13
Ultrasound screening				
0	39	51	45	43
1	35	31	29	33
2	19	16	23	19
3+	7	2	3	5
Health education on nutrition given	77	71	84	77
Health education on baby care given	71	70	86	73
Health education on maternal care given	83	79	95	84

### Delivery and postnatal care

Most of the women gave birth in public health facilities, but 12% of the women gave birth in a private clinic or at home (Table 5). A total 8% of deliveries were caesarean sections, of which more than 90% were performed in county level hospitals. There was an association between frequencies of prenatal visits and the place of delivery (Table 2). The women who delivered in public facilities had on average almost twice as many prenatal visits as the women who delivered in private clinic or at home. A total of 35% of women who delivered outside of public health facilities had not received prenatal care at all. In comparison, 5% of the women who delivered in public health facilities had not used prenatal care.

**Table 5:** Place of delivery by parity (%).

	County hospitals	Township hospitals	Private clinics	At home	Total
Parity 1 (n = 450)	27.9	59.7	10.0	2.4	100.0
Parity 2+ (n = 139)	21.2	63.7	10.9	4.3	100.0
Parity unknown (n = 110)	19.2	72.7	4.5	3.6	100.0

Total (n = 699)	25.0	62.7	9.3	3.0	100.0

According to interviews of the township hospital managers and township midwives, most of the women left the hospital within 24 hours of the delivery; the women only made postnatal visits to the clinic if the baby or the woman herself was sick; none of the midwives made postnatal home visits.

## Discussion

Prenatal care in the study area was generally available in every township: there was a midwifery department in every township hospital with at least one midwife, though the equipment was not always adequate. However, the public facilities and resources were under-used in terms of timing and frequency of prenatal care. Further, the content of care did not follow government recommendations: some simple procedures were not carried out, while prenatal ultrasound testing in pregnancy was becoming more frequent than might be medically necessary. The document "Management method of systematic maternal care in rural areas" issued by the Chinese Ministry of Health (MOH) stipulated that the pregnant women should have their first prenatal visit before 13 weeks of gestation and have at least 5 prenatal visits and at least 3 postnatal visits (within 42 days of delivery) [24].

In the study area, only about half of pregnant women complied with MOH requirement on timing and frequency of prenatal care visit. Furthermore, no women complied with the recommended postnatal visits, either by women to clinics or by midwives to mother's homes. The Chinese recommendation for the frequency of postnatal visits was higher than that of WHO [27]. Despite the low number of prenatal visits, hospital deliveries were common, the rate almost reaching the level of urban areas of China [19].

Given the local family planning policy – a farmer couple is permitted to have another child if their first child is a girl – and the son preference in the study area [25], it was surprising that there was no difference in the timing or number of prenatal visits or hospital deliveries by parity. Reported ultrasound screening was not more common in higher parities. To explain these findings, further data would be needed.

The proportion of pregnant women who had their first prenatal care visit before 13 weeks gestation in study area was lower than the average level for rural areas nationally (55%). The proportion of women who had more than 4 prenatal visits was a bit higher (55%) than the average level of rural areas (36%) at the same period of time [7].

The Ministry of Health (MOH) recommended that pregnant women with serious condition should be referred to county level hospitals. The MOH norms for systematic maternal care stipulated that the county, township, and village networks of prenatal care system should be set up by the local government, but did not state where the financial resources should come from.

According to the county regulation, private practitioners were not permitted to provide delivery services, but they could be involved in prenatal care. However, one in six women sought delivery care from private practitioners.

The Minister of Health recommended that the pregnant woman could take an ultrasound test after 28 weeks of gestation if medically necessary. It was very likely that many women received an ultrasound before 28 weeks of gestation because the study showed that as many as 58% of women had received at least one ultrasound scan.

Previous studies in many underdeveloped or poor rural areas of China have shown that expensive user fees, long travelling distances or a lack of transportation were the most important obstacles to gaining access to available prenatal care [28,29]. In this study, the most important factor reported as a hindrance to prenatal care by the women was their lack of recognition of the importance of prenatal care. While a lack of time and transportation were mentioned as reasons for not seeking prenatal care, by the far the most important reason given was a perception of the uselessness and insignificance of prenatal care. Two possible reasons may explain this finding: either the women did not accept the importance of the prenatal care in preventing adverse events, or they recognized the importance but did not believe that the local health services could deliver care adequately. The lack of basic equipment in the health facilities supports the latter belief. Further the proportion of women who did not seek prenatal care was higher among those who gave birth in the private clinics. Some of these women might have wanted to have private delivery care because of the illegal status of their pregnancy [30]. Due to the sensitivity of the family planning policy, this issue could not be directly discussed with the women.

The equipment in the prenatal care facilities in townships did not match the needs of good prenatal care. Rather it seems that investments in equipment were based on the ability to generate income for the hospital. In terms of providing prenatal care, weighing scales would be a priority as they are cheap, readily available in the market and important for the monitoring of every pregnancy. In contrast, the ultrasound machine is of less importance at the primary care level: it is useful for a lesser number of patients and is more expensive. However, more township hospitals owned ultrasound machines than weighing scales and height-measuring equipment. Apparently, women were either encouraged to use the ultrasound machine, or the women asked for scans themselves.

The consultations were almost always free for pregnant women. There was little incentive to adequately equip hospitals for routine prenatal care as it generated no extra income for the hospital. Hospitals profit from laboratory tests and birth services, so these services were promoted by the hospital. The high hospital delivery rate may also be attributed to the "Healthy mother and well baby" campaign launched by the health-related international organizations such as WHO and UNICEF and supported by the Chinese Government since the early 1990s [31,32].

The Chinese Ministry of Health and local health authorities had set requirements for conducting prenatal care and recommendations for its content, but had not offered funding or guidance on how to fund preventive activities. If township hospitals conducted systematic maternal care with little or no payment, they would not generate enough income to support the hospital. In rural areas of a western province, a new Co-operative Medical Scheme has been initiated. It involves risk sharing among farmers, financed jointly by individual farmers and local and central government [33,34]. If the scheme is extended to cover maternal care, it might be one solution to the current problems of financing prenatal care.

Because of the decentralization of state administration, policies or regulations from a high level of government have less impact on lower level government. In our study, this was seen in the lack of authority the county health bureau had over township hospitals. Their management decisions were made from an economic standpoint, rather than from a medical view; that is, balancing the income and expenses of the facilities was more important than complying with accepted medical recommendations and regulations. This management model has been questioned by many researchers [35,36], but is also still defended by some [37]. From the point of view of the quality of health care, health policies should be supported with financing schemes that do not work against the intent of the policy.

### Strengths and weaknesses of the study

Few studies describing maternal care in China in detail are published in English [3,9,10,12]. This is the first paper aimed toward an international audience that explores the organization, provision and use of maternal care in rural China, while covering both service providers and users.

One drawback of the study is that only one county was studied, and the results may not be applicable to other areas. The second limitation of our study is that study townships were purposely selected and did not cover townships without a township hospital. The third limitation is that mothers with dead infants were not included in the study. The second and third limitations might result in overestimation of the maternal care use in the study county. For example, the hospital delivery rate in the townships without hospital might be lower than that in townships with a hospital. The number of stillborn children was small, and can not effect the study results much.

The fourth limitation is that even though our description covers various elements of maternal care, it still lacks important details, such as the detailed content of pre- and postnatal care. The interviews of the women were conducted by local health workers, which may have facilitated them because they came from the same area as the interviewees. However, women may have perceived that they represented health authorities, which might have decreased women's willingness to speak freely in interviews. We do not know whether this decreased the reliability of the results, and if so, to what direction.

Further, to do the study required collaboration with the county health officials. As a result, we were not allowed to visit or interview private practitioners, because the government officials thought this to give credit to that illegal activity; private provision of delivery care was declared illegal in the county. Studying private delivery care might have been interesting taking into account the differences in prenatal care usage between women delivering in private and public facilities.

## Conclusion

Delivery care was better accepted by women and more systematically provided than prenatal and postnatal care in the study area. Reliance on user fees gave the hospitals an incentive to put more emphasis on revenue generating activities such as delivery care instead of prenatal and postnatal care.

## Competing interests

The author(s) declare that they have no competing interests.

## Authors' contributions

ZW was involved in the conception and design of the research idea, coordination and acquisition of the data, analyses and interpretation of the data, and in drafting, revising the manuscript. KV was involved in the conception and design of the research idea, analyses and interpretation of the data, and in drafting, revising the final manuscript; XL was involved in the coordination and acquisition of the data, analyses and interpretation of the data, and in revising the manuscript. EH was involved in the conception and design of the research idea, analyses and interpretation of the data, and in drafting, reviewing and revising the manuscript.

All authors read and approved the final manuscript.

## Pre-publication history

The pre-publication history for this paper can be accessed here:


